# Personalized neoantigen vaccine enhances the therapeutic efficacy of bevacizumab and anti-PD-1 antibody in advanced non-small cell lung cancer

**DOI:** 10.1007/s00262-023-03598-x

**Published:** 2024-01-27

**Authors:** Xiuhua Lin, Shichuan Tang, Yutong Guo, Ruijing Tang, Zhenli Li, Xinting Pan, Geng Chen, Liman Qiu, Xiuqing Dong, Ling Zhang, Xiaolong Liu, Zhixiong Cai, Baosong Xie

**Affiliations:** 1 Shengli Clinical Medical College of Fujian Medical University, Fuzhou, China; 2 The United Innovation of Mengchao Hepatobiliary Technology Key Laboratory of Fujian Province, Mengchao Hepatobiliary Hospital of Fujian Medical University, Fuzhou, China; 3 The Liver Center of Fujian Province, Fujian Medical University, Fuzhou, China; 4 Department of Pulmonary and Critical Care Medicine, Fujian Provincial Hospital, Fuzhou, China; 5 Academy of Integrative Medicine, Fujian University of Traditional Chinese Medicine, Fuzhou, China; 6 Fujian Key Laboratory of Integrative Medicine On Geriatrics, Fujian University of Traditional Chinese Medicine, Fuzhou, China

**Keywords:** Non-small cell lung cancer, Neoantigen vaccine, Chemotherapy-free treatment, Ki67-CD8^+^ T cells

## Abstract

Clinically, a considerable number of non-small cell lung cancer (NSCLC) patients are unable to receive or resist chemotherapy, and the efficacy of non-chemotherapy treatment strategies based on anti-angiogenic agents combined with immune checkpoint blockade is still unsatisfactory. Neoantigen vaccine, based on personalized tumor DNA mutations, could elicit tumor specific T cell infiltration into the tumor site, exerting potent anti-tumor efficacy. Here, we evaluated the feasibility and safety of a new antitumor strategy by adding neoantigen vaccine to the regimen of bevacizumab and anti-PD-1 antibody. Firstly, 7 novel immunogenic neoantigen peptides were identified and developed for neoantigen vaccine (LLCvac), which can elicit strong antitumor immune response in vivo. Then, in orthotopic lung cancer model, LLCvac further combining with bevacizumab and anti-PD-1 antibody exerted a stronger antitumor effect, exhibiting significant decrease of tumor volume without obvious toxicity. Furthermore, tumor immune microenvironment assessment also showed that the proportion of neoantigen-specific T cells in blood could be induced dramatically by the combined therapy. And a large amount of neoantigen-specific Ki67-positive CD8^+^ T cells were found in tumor tissues, which infiltrated tumor tissues effectively to kill tumor cells expressing identified neoantigens. Overall, these results suggested that this combined therapy could safely induce robust antitumor efficacy, serving as an effective chemotherapy-free strategy for NSCLC treatment.

**Supplementary Information:**

The online version contains supplementary material available at 10.1007/s00262-023-03598-x.

## Introduction

Lung cancer is the malignance tumor with the highest mortality rate worldwide, and its incidence trend is becoming younger in recent years [1]. Combined therapy based on chemotherapy is the first-line treatment for advanced non-small cell lung cancer (NSCLC) patients without actionable driver gene mutations. In the KEYNOTE-021 trial, the objective response rate for the combination of chemotherapy and immunotherapy (pembrolizumab) in advanced lung cancer has reached 55% [2]. However, due to the significant toxicity and side effects of chemotherapy, a considerable proportion of patients are unable to tolerate chemotherapy or have significant resistance to chemotherapy, particularly in the elderly or immunocompromised populations [3]. Clinically, the combined treatment strategy without chemotherapy for advanced NSCLC patients mainly includes anti-angiogenic therapy and immunotherapy, such as bevacizumab (BEV) plus immune checkpoint inhibitors (ICIs) [4]. However, their efficacy is still inferior to the combined therapy based on chemotherapy [5]. One of the main reasons is that after anti-angiogenesis and immunotherapy, most of the T cells that infiltrate into tumor tissue are not tumor-specific. [6]. Therefore, how to improve the infiltration of tumor-specific lymphocytes without significantly increasing toxicity and side effects is a key strategy to improve the efficacy of chemotherapy-free treatment.

Tumor neoantigens, belonging to tumor-specific antigen, are a class of immunogenic polypeptides produced by gene alterations during tumor development [7, 8]. They can be bound to the major histocompatibility complex (MHC) and further presented on the surface of tumor cells, thereby activating immune cells for tumor infiltration to enhance tumor immunotherapy [9, 10]. Therefore, therapeutic vaccines based on tumor neoantigens have the advantages of high specificity and less toxicity and side effects, and can be used for precise tumor immunotherapy. Previous studies by us and other teams have demonstrated that tumor neoantigen vaccine can induce tumor-specific lymphocyte infiltration in several solid tumors, including lung and liver cancer, and exert potent anti-tumor effect [11–14]. However, the immunosuppressive microenvironment (such as angiogenesis and high expression of PD-L1 in the tumor microenvironment) will still significantly inhibit the antitumor activity of tumor-specific T cells induced by neoantigen vaccine. Therefore, the design of neoantigen vaccine-based combination therapy to achieve full anti-tumor efficacy is still under investigation. Recently, the combination of neoantigen vaccine with other first-line treatment options to improve efficacy has emerged as an available option [15, 16]. In a phase 1b clinical trial, combining the personalized vaccine NEO-PV-01 with chemotherapy and pembrolizumab in first-line metastatic NSCLC has shown to be safe and well tolerated and induced neoantigen-specific CD4^+^ T cell responses with an effector phenotype [15]. However, for the chemotherapy-free combined therapy strategy, whether the neoantigen vaccine can exert its strong anti-tumor response to enhance the therapeutic efficacy of antiangiogenic therapy and immunotherapy (such as Bev and anti-PD-1 antibody), as well as the potential mechanisms remain to be classified.

Here, we firstly identified high immunogenic tumor specific neoantigen from mouse Lewis lung carcinoma cell line (LLC) based on whole exome and transcriptome sequencing, and further constructed a novel personalized NSCLC neoantigen vaccine for evaluating its potential antitumor efficacy in subcutaneous and orthotopic lung cancer mouse models. Meanwhile, incorporating neoantigen vaccine in Bev and anti-PD-1 antibody as a triple combination therapy could significantly improve the antitumor immune response of NSCLC without significantly enhancing side effects. Overall, this study will provide a new therapeutic strategy with high efficacy and high safety for the chemotherapy-free treatment of NSCLC.

### Methods

#### Cell line culture and authentication

LLC cells were obtained from BNCC (BeNa Culture Collection, China) and cultured in DMEM (Gibco) medium supplemented with 10% FBS at 37 °C with 5% CO_2_. This cell lines were subjected to a complete validation process and were authenticated by performing Short Tandem Repeat (STR) profiling analysis at the past one years. Before experiments, the cell line cultures were subjected to determine for mycoplasma-free status by using a PCR Mycoplasma detection kit (MycoSEQ Mycoplasma Detection Kit, Thermo Fisher Scientific).

### Neoantigen identification and immunogenicity validation

To identify potential neoantigens derived from LLC tumors, we performed whole exome sequencing and transcriptomic sequencing on the LLC cell line and C57BL/6 mouse tail tissue. The detailed methods of neoantigen identification were shown in online supplemental methods and previously published literature [17]. Then top 20 potential neoantigen mutations were selected and prioritized for long peptide synthesis (17 amino acids in length) using standard solid-phase synthetic peptide chemistry (> 95% purity, Jinsirui Biotechnology, China). For neoantigen immunogenicity validation, 16 successfully synthesized candidate neoantigen peptides were randomly divided into two pools (100 µg/peptide) and mixed with 50 µl Poly(I:C) (Guangdong South China Pharmaceutical), then injected subcutaneously in the lateral flank of 2 groups of C57BL/6 mice on days 0, 4 and 8, respectively. Mice were sacrificed on day 14 and splenic T cells were harvested for ex vivo interferon (IFN)-γ ELISPOT assay according to the manufacturer's instructions. The detailed methods of ELISPOT assay were shown in online supplemental methods.

### In vivo tumor models and evaluation of anti-tumor efficacy

All tumor challenges were performed by injecting LLC cells into 6–8 weeks old female C57BL/6 mice. The subcutaneous lung cancer model was established by subcutaneous injection of 2 × 10^6^ LLC cells into the right axilla. An orthotopic lung cancer model was established by direct injection of 1 × 10^6^ LLC cells via the tail vein. Tumor monitoring of subcutaneous lung cancer model was performed by a single operator every three days in two dimensions using calipers. Tumor volume was calculated from the measured data as follows: V = AB^2^/2 (A is the long diameter and B is the short diameter). When the tumor volume grew to 50–80 mm^3^ recorded as day 0, the mice were randomly divided into 4 groups (n = 6) to start treatment. In the experimental group, a neoantigen peptide vaccine (LLCvac) consisting of 7 identified neoantigen peptides (100 µg/peptide/mouse) combined with an optimized clinically available dual immune adjuvants [50 µg Poly(I:C) + 50 µg thymosin alpha-1(ZadaxiN, Sciclone pharmaceuticals, USA)] was administered subcutaneously to the tumor-bearing mice starting on day 0, once every four days for three times. The control groups were treated for the same duration, and the injected drugs were phosphate buffered saline (PBS), neoantigen peptide alone, and Poly(I:C) + thymosin alpha-1, respectively.

For the orthotopic lung cancer model, mice were randomly divided into 4 groups (n = 6) after 8 days of tumor inoculation (recorded as day 0) and then the treatment was initiated. LLCvac was administered in the same manner as the subcutaneous lung cancer experimental group. Tumor-bearing mice were injected intraperitoneally with bevacizumab (Bev: 5 mg/kg, 100 µg/mouse, Roche Diagnostics GmbH, Germany) and tail vein with anti-PD-1 antibody (2.5 mg/kg, 50 µg/mouse, Leinco Technologies, USA) (anti-PD-1 + Bev) or in combination (LLCvac + anti-PD-1 + Bev) twice weekly for 2 weeks.

At the end of treatment, mice were sacrificed by cervical dislocation and tumor volume was recorded for subcutaneous cancer, and the number of tumor nodules in the lung was recorded for orthotopic carcinomas, respectively. In addition, lymph nodes, spleen and tumor tissues were isolated and prepared into single cell suspensions for flow cytometry analysis. A portion of the tumor tissue was excised for formalin-fixed paraffin embedding using the Swiss-rolling method, followed by immunofluorescence and immunohistochemistry. The detailed methods were shown in online supplemental methods.

### Single-cell RNA sequencing of LLC tumor tissue

Fresh tumor tissues were collected from LLC mouse models treated with LLCvac and/or BeV plus anti-PD1 antibody for 20 days, as well as a PBS-treated control group. The tissues were immediately placed on ice and digested with 2 mL of sCelLiveTM Tissue Dissociation Solution (Singleron, China). The resulting cells were then suspended in PBS. Cell viability was assessed microscopically using the trypan blue exclusion test (Gibco, Grand Island, NY, USA), and samples with cell viability greater than 80% were considered suitable for subsequent experiments. The cell suspension was further diluted in PBS to achieve a concentration of 3 × 10^5 cells/ml. Single cell isolation and mRNA capture were performed using the Singleron Matrix Single Cell Processing System (Singleron, China). Subsequently, scRNA-seq libraries were constructed by reverse transcription, amplification, and library construction using the GEXSCOPE Single Cell RNA Library Kit (Singleron, China). Only libraries that met quality standards were sequenced on the Illumina HiSeq 6000 platform (150 bp paired-end reads). The detailed data analysis methods were shown in online supplemental methods.

### Statistical analysis

Pearson’s correlation coefficient was used to evaluate the correlation matrices. Sample data were compared using two-tailed Student’s t-test or one-way analysis of variance using GraphPad Prism V.16.0 software, *p < 0.05 was considered statistically significant. **p < 0.01, ***p < 0.001, ****p < 0.0001. All data were presented as means ± SD/SEM of at least three biologically independent samples.

## Results

### Neoantigen identification and immunogenicity evaluation for mouse lung cancer

**Fig. 1:**
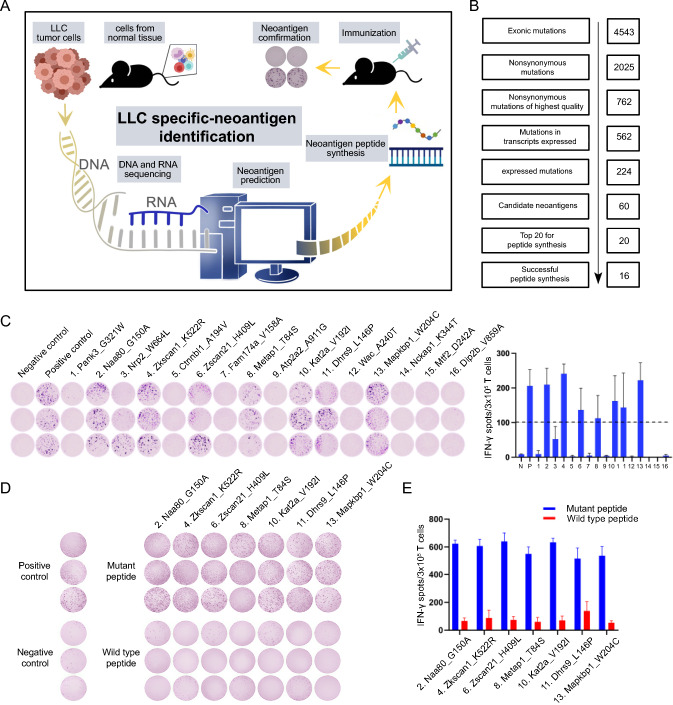
Neoantigen identification and immunogenicity evaluation. **A** Tumor neoepitope identification processes for LLC cells. **B** Screening workflow of neoantigen peptides. **C** Potential neoantigen immunogenicity validation performed by ELISPOT assay. 7 neoantigens with more than 100 spots were selected to develop neoantigen vaccine indicated by dotted line. **D** Cross-reactivity analysis between seven identified neoantigen peptides and corresponding wild-type peptides. **E** The quantitative histogram of cross-reactivity analysis of ELISPOT assay (n = 3), the data are presented as the mean ± SD

To prepare tumor neoantigen vaccines for lung cancer cell lines, we first performed whole exome and transcriptome sequencing of LLC cells and C57BL/6 mice tail to identify potential neoantigen mutations (Fig. 1A). As shown in Fig. 1B, a total of 762 individual LLC somatic mutations were identified with high quality when compared to the C57BL/6 genome, and 224 of them were further found to be expressed at the RNA level by LLC transcriptome data analysis (variant allele frequency ≥ 10%, read depth ≥ 20 and transcripts per million of the corresponding gene ≥ 1). Based on this, we further predicted the binding affinity between these mutations and MHC I (LLC cells and C57BL/6 mice: H-2 K^b^ allele) by eight MHC binding affinity prediction algorithms (NetMHCpan, NetMHC, NetMHCcons, PickPocket, MHCflurry, SMM, SMMPBMC and MHCnuggetsI). These data showed that 60 mutations producing short peptides with high binding affinity to MHC (H-2 K^b^ allele, median binding affinity percentile rank across all MHC binding affinity prediction algorithms <  = 2%) were predicted, and considered as potential tumor neoantigen mutations.

The mutated long peptides (17aa) derived from the top 20 potential neoantigen mutations were synthesized for immunogenicity evaluation. After that, 16 successfully synthesized candidate neoantigen peptides were randomly divided into 2 pools and mixed with immune adjuvant Poly(I:C) for subcutaneous immunization of C57BL/6 mice at the lateral flank on day 0, day 4 and day 8, respectively (Table S1). Then, these immunized mice were sacrificed at 14 days after initial immunization, and the splenic T cells were isolated for ex vivo interferon (IFN)-γ ELISPOT assay. As shown in Fig. 1C, 7 out of 16 synthesized mutated long peptides (Naa80_G150A, Zkscan1_K522R, Zscan21_H409L, Metap1_T84S, Kat2a_V192I, Dhrs9_L146P, Mapkbp1_W204C) could induce robust immune responses in T cells stimulated by each mutated long peptide-pulsed autologous matured dendritic cells (DCs). To further characterize whether these mutant long peptides have immune cross-reactivity with their corresponding wild type peptides, we formed a pool of them and mixed with Poly(I:C) to immunize and treat mice as described above, and then mutant peptides and corresponding wild-type peptides were pulsed with autologous DCs respectively for downstream ELISPOT analysis. As expected, these 7 neoantigen peptides have no significant immunogenic cross-reactivity compared with the wild type (Fig. 1D). These results suggested that these 7 peptides could elicit strong targeted immunogenicity in C57BL/6 mice and can be identified as neoantigens for LLC.

### Antitumor efficacy evaluation of Lung cancer neoantigen vaccine

**Fig. 2:**
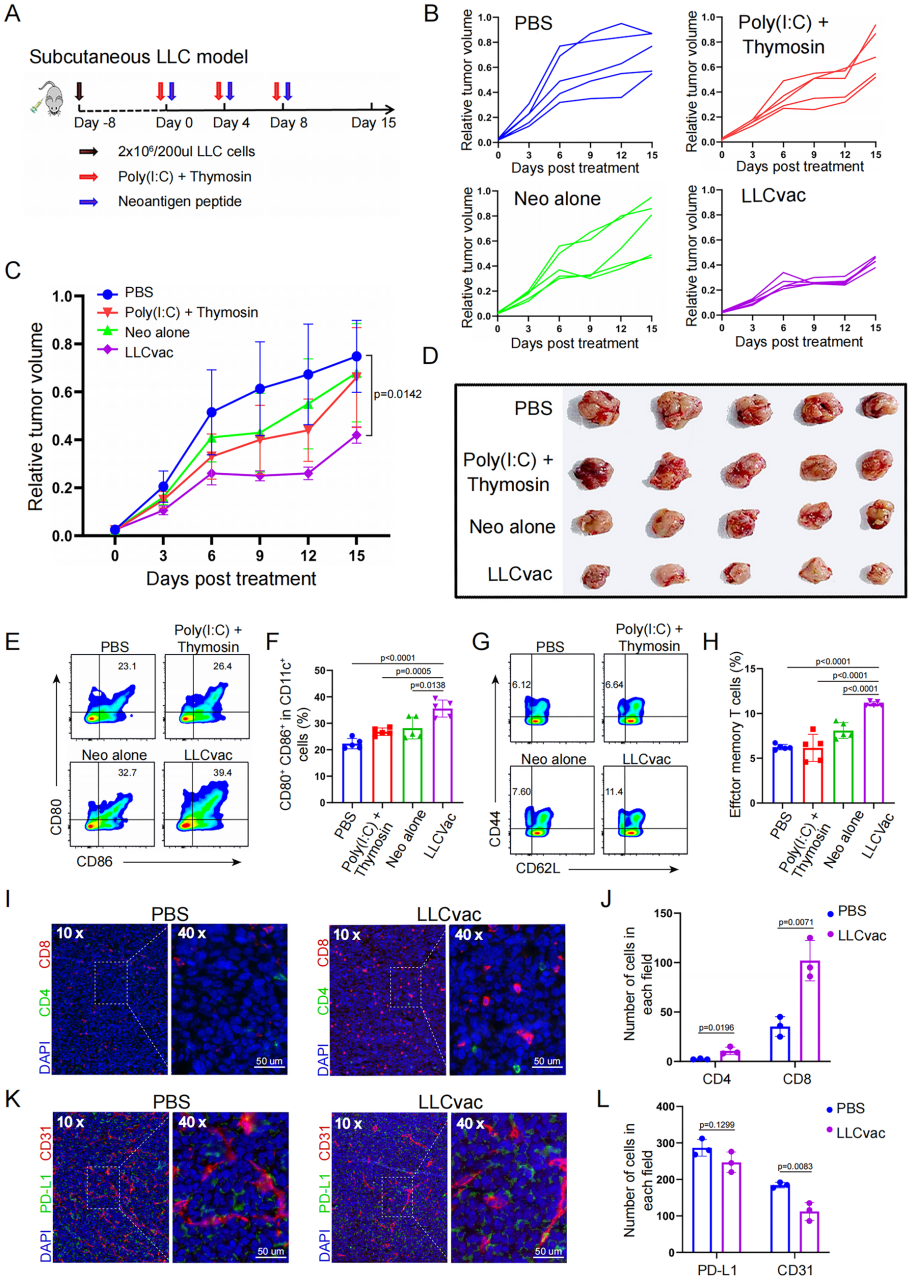
Antitumor efficacy of LLCvac in subcutaneous LLC model. **A** Schematic diagram of vaccination-associated therapy in subcutaneous LLC model. **B** and **C** Tumor volume monitoring in LLC-tumor-bearing C57BL/6 mice (n = 5) after received with PBS, Poly(I:C) + Thymosin alpha-1, Neoantigen peptides alone, or LLCvac for three times administration, respectively. **D** Photographs of tumors excised from mice after 15 days of treatments as indicated. **E** and **F** The percentage and statistic analysis of matured DCs with CD80 and CD86 co-expression in the lymph nodes detected by flow cytometry (n = 5). **G** and **H** The percentage and statistic analysis of effector memory T cells in splenic CD8^+^ T cells detected by flow cytometry. **I** and **J** The represent graph and statistic analysis of inflating CD4^+^ and CD8^+^ T cell in LLC tumor after LLCvac treatment (n = 5). **K** and **I** The represent graph and statistic analysis of angiogenesis and PD-L1 expression in LLC tumor after LLCvac treatment (n = 3). The data are presented as the mean ± SD

To further investigate the anti-tumor activity of neoantigen peptides, these 7 neoantigen peptides were further mixed with an optimized clinically available dual immune adjuvant [Poly(I:C) and thymosin alpha-1] to prepare personalized neoantigen vaccine for LLC (LLCvac). Firstly, subcutaneous mouse model of lung cancer was established by subcutaneous injection of 2 × 10^6^ LLC cells into the flank of mice, and further randomly divided into 4 groups to receive PBS (control group), immune adjuvant alone [Poly(I:C) plus thymosin alpha-1], neoantigen peptide alone (a pool containing 7 neoantigen peptides) and neoantigen vaccine (LLCvac) on days 0, 4, and 8 respectively (Fig. 2A). As shown in Fig. 2B–D, after 15 days of initial immunization, LLC tumor treated with PBS group grew rapidly; meanwhile, the mice treated with immune adjuvant or neoantigen peptides alone showed limited anti-tumor activity for LLC tumor. Obviously, the growth rate of subcutaneous LLC tumor was significantly slowed down after LLCvac treatment (*p* = 0.0142, t-test between PBS group and LLCvac group). These findings showed that LLC tumor-targeted neoantigen vaccine was successfully prepared and could induce potent anti-tumor activity in subcutaneous mouse model of lung cancer.

To further investigate whether LLCvac exerted anti-tumor activity by activating the systemic immune responses, we isolated the subcutaneous lymph node cells and spleen cells of the treated mice as indicated to evaluate the maturity of lymph node DC and the amount of immune memory T cells in the spleen by flow cytometry. As shown in Fig. 2E–H, compared with the other three groups, both the maturity of lymph node DC (CD80^+^ and CD86^+^) and the amount of spleen immune memory T cells (CD44^+^ and CD62L^−^) were significantly increased in the LLCvac group respectively (*p* < 0.0001 and *p* < 0.0001, t-test between PBS group and LLCvac group), indicating that neoantigen vaccine successfully activated the immune system. Moreover, multicolor immunofluorescence staining of CD4 and CD8 antibodies was further performed in tumor tissue to assess the infiltration of T cells into the tumor immune microenvironment. As expected, both CD4^+^ T cells and CD8^+^ T cells were significantly enriched in tumor tissue from mice treated with LLCvac (*p* = 0.0196 and *p* = 0.0071, Fig. 2I, J).

Meanwhile, we also noticed that LLCvac could only slow down the growth rate of lung cancer, but not shrink the tumor volume. Since angiogenesis and upregulation of immune checkpoint expression are the key factors for tumor suppression or resistance to immunotherapy, we further investigated angiogenesis and PD-L1 expression in tumor tissues after treatment with neoantigen vaccines. As shown in Fig. 2K, L, the levels of angiogenesis and PD-L1 expression in the LLC tumor microenvironment remained high, although there was a certain decrease after LLCvac treatment. Therefore, we sought to investigate whether neoantigen vaccines could be further combined with Bev and anti-PD-1 antibody to build novel chemotherapy-free treatment strategy to improve the therapeutic efficacy of NSCLC.

### Anti-tumor efficacy and the safety of novel combined therapy

**Fig. 3:**
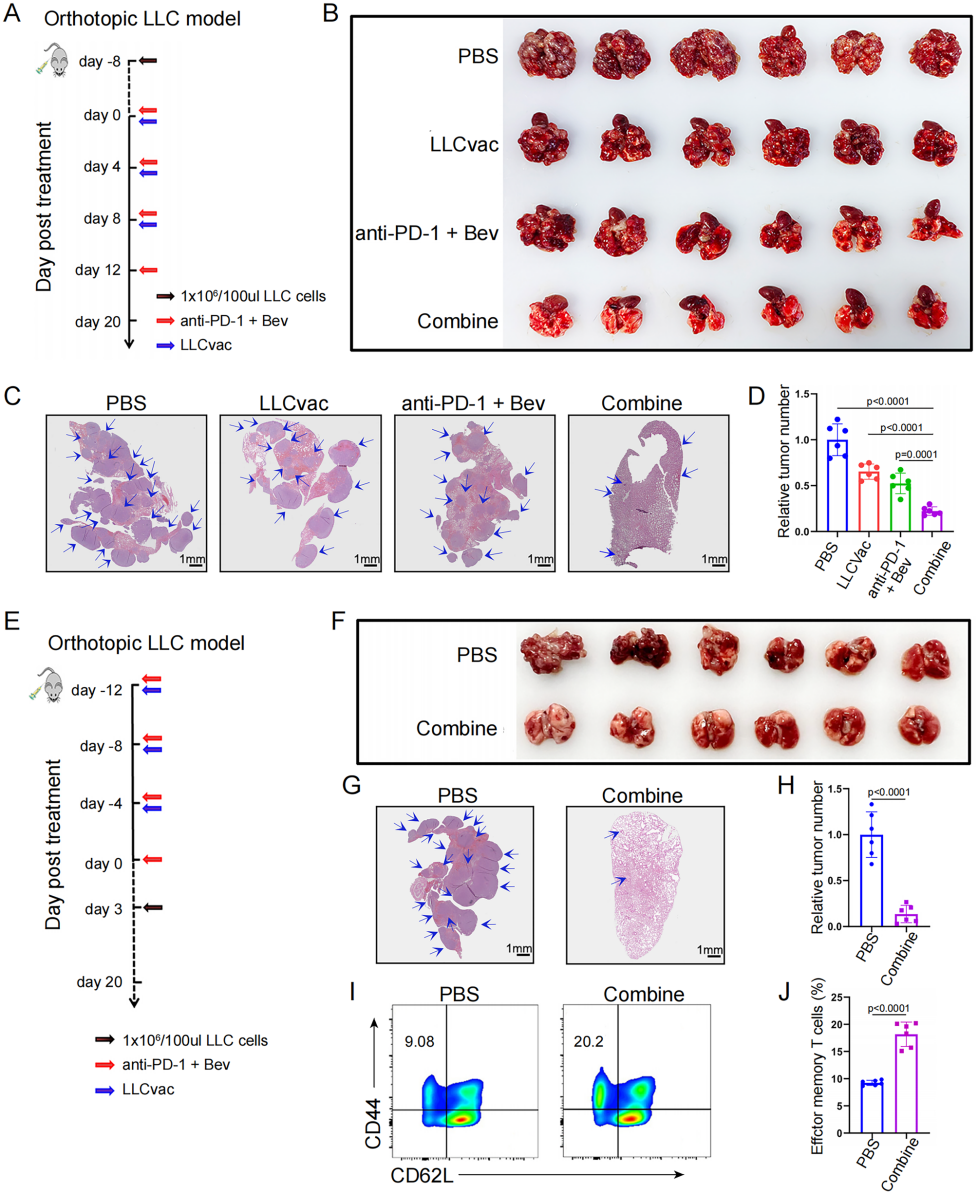
Evaluation the antitumor effects under the combination treatment of LLCvac and anti-PD-1/bevacizumab in orthotopic LLC model. **A** Schematic diagram showing the timeline of orthotopic LLC model construction and treatment. **B** Photographs of tumor-bearing lungs collected from mice after different treatments (PBS, LLCvac, anti-PD-1 + Bev, or LLCvac + anti-PD-1 + Bev named as combine) (n = 6). **C** and **D** The H&E staining and statistic analysis of tumor nodules from lung tissues as indicated. The blue arrow indicated one tumor nodule, which has clear boundary with non-tumor sites (n = 6). **E** Schematic diagram showing the timeline of orthotopic LLC model construction for tumor prevention and treatment. **F** Photographs of tumor-bearing lungs collected from mice after treatments as indicated (n = 6). **G** and **H** The H&E staining and statistic analysis of tumor nodules from lung tissues as indicated. The blue arrow indicated one tumor nodule, which has clear boundary with non-tumor sites. **I** and **J** The percentage statistic analysis of effector memory T cells in splenic CD8^+^ T cells as indicated (n = 6). The data are presented as the mean ± SD

To better mimic the immune microenvironment of NSCLC, an orthotopic lung cancer mouse model was established by tail vein injection of LLC cells, which could specifically induce LCC cells to grow on lung tissue in the form of nodules. As shown in Fig. 3A, the established orthotopic lung cancer models were randomly divided into four groups (n = 6) and further respectively received treatment with PBS (control group), LLCvac vaccination (subcutaneous injection), anti-PD-1 antibody (intravenous caudal injection) and Bev (intraperitoneal injection), and combined treatment strategy (LLCvac + anti-PD-1 + Bev). Then, 20 days after initial treatment, those mice were sacrificed, and the whole lung tissue was isolated and photographed to evaluate the anti-tumor efficacy. As shown in Fig. 3B, all lung tissues collected from PBS-treated mice were overgrown with tumor. The neoantigen treatment group, similar to the above treatment results in the subcutaneous tumor model, has some therapeutic effect compared to PBS treatment, but all tumors are still progressing. Interestingly, the anti-tumor efficacy of anti-PD-1 and Bev therapy is better than that of the previous two groups, and has strong anti-tumor efficacy, which is consistent with clinical performance. However, the therapeutic effect of LLC tumor in anti-PD-1 and Bev therapy still needs to be further improved. Surprisingly, when the orthotopic LLC model mice treated with LLCvac plus anti-PD-1 and Bev, the anti-tumor efficacy in lung tissue was significantly improved compared to the other three groups (p = 0.0001). The number and sizes of tumor nodules stained in lung tissue HE sections from the different treatment groups further confirmed the significant anti-tumor efficacy of LLCvac plus with anti-PD-1 and Bev (Fig. 3C, D). These results implied that combined treatment could significantly improve the anti-tumor efficacy in orthotopic lung cancer mouse model and prolong survival. To further evaluate the preventive capacity of the potential tumor recurrence and metastasis for the combined therapy, the C57BL/6 mice were first treated with the combined therapy as indicated and then injected with 1 × 10^6^ LLC cancer cells by tail intravenous injection after 3 days (Fig. 3E). As expected, the mice treated with PBS (control) suffered from rapid tumor growth, while the mice treated with combined therapy completely delayed tumor growth (Fig. 3F–H) and exhibited more effector memory T cells in spleen (Fig. 3I, J).

As a suitable synergistic combined therapy strategy, in addition to better anti-tumor efficacy, lower toxicity and side effects should also be emphasized. Therefore, we further evaluated the changes of biochemical indicators in mice receiving combined treatment. As shown in Fig. S1, the combined treatment group did not induce obvious abnormalities in liver and kidney function. These results suggested that neoantigen vaccine plus anti-PD-1 and Bev is a kind of novel strategy with feasibility and safety for chemotherapy-free treatment of NSCLC.

### Combined treatment induces immune responses in orthotopic LLC model

**Fig. 4:**
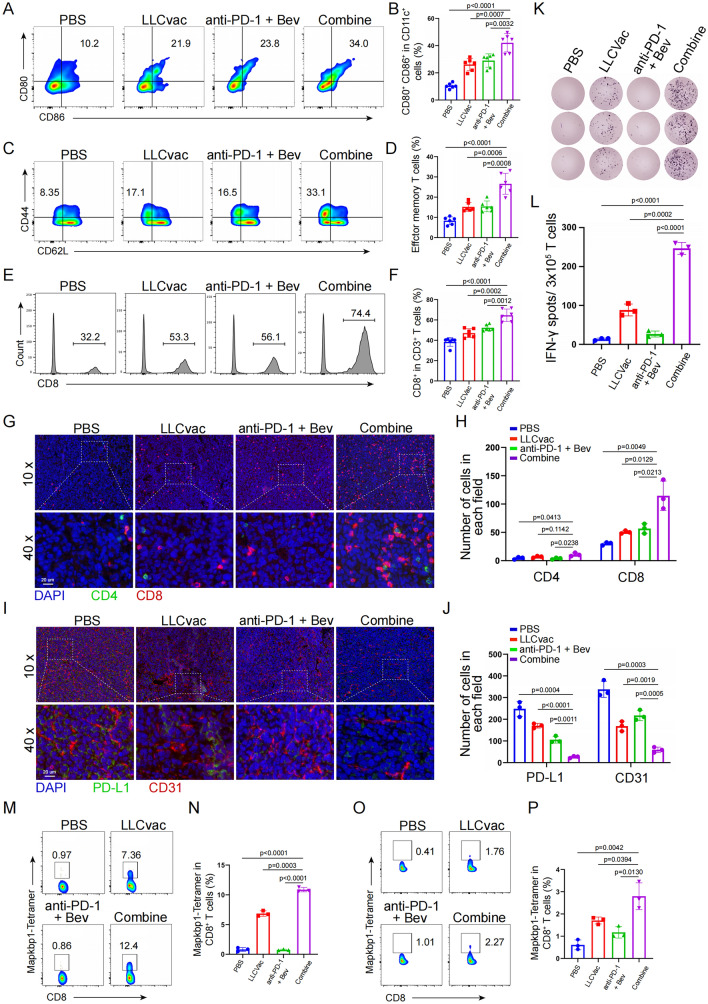
Immune response induced in orthotopic LLC tumor-bearing mice after combined treatment. **A** and **B** The percentage and statistic analysis of matured DCs with CD80 and CD86 co-expression in the lymph nodes collected from different treatment as indicated by flow cytometry (n = 6). **C** and **D** The percentage and statistic analysis of effector memory T cells in splenic CD8^+^ T cells collected from different treatment as indicated by flow cytometry (n = 6). **E** and **F** the infiltration percentage and statistic analysis of CD8^+^ T cells in tumor tissue collected from different treatment as indicated by flow cytometry (n = 6). **G** and **H** Immunofluorescence staining showing the represent graph and statistic analysis of the infiltrating CD4^+^ and CD8^+^ T cells in tumor tissue collected from different treatment as indicated (n = 3). **I** and **J** Immunofluorescence staining showing the represent graph and statistic analysis of the angiogenesis and PD-L1 expression in tumor tissue collected from different treatment as indicated (n = 3). **K** and **L** ELISPOT assay showing neoantigen-specific reactivity of splenic T cells against 7 neoantigen peptides on day 20 after treatment (n = 3). The percentage and statistic analysis of Mapkbp1-specific CD8^+^ T cells in PBMCs (**M** and **N)** and tumor tissues (**O** and **P**) collected from different treatment as indicated, respectively (n = 3). All samples analyzed here were from Fig. 3A mouse model. The data are presented as the mean ± SD

To further classify potential immune responses in the orthotopic lung cancer mouse model treated with combined therapy, the status of several immune cell phenotypes including mature DCs (CD11c^+^) in lymph nodes (LNs) and effector memory CD8^+^ T cells (*T*_Ems_, CD44^+^ and CD62L^−^) in spleen were evaluated. As shown in Fig. 4A–D, the percentages of mature DCs in LNs and CD8^+^ T_Ems_ in spleen from mice treated with combined therapy were significantly higher than those in the other three treated groups. Meanwhile, the infiltration of effector CD8^+^ T cells in tumor tissue were further assessed by flow cytometry. As expected, the proportion of effector CD8^+^ T cells in tumor infiltrating CD3^+^ T cells was significantly upregulated in the combination treatment group (Fig. 4E, F). This phenomenon was also confirmed by multicolor immunofluorescence staining of CD4 and CD8 antibodies in tumor tissues (Fig. 4G, H). Monitoring of angiogenesis and PD-L1 expression by immunofluorescence staining also showed that the number of angiogenesis and the expression level of PD-L1 in the tumor microenvironment were also significantly reduced after combined treatment, which positively correlated with therapeutic efficacy in orthotopic lung cancer models (Fig. 4I, J).

The neoantigen-specific reactivity of splenic T cells against 7 neoantigen peptides was then assessed by ELISPOT analysis. As expected, the number of IFN-γ spots induced by the neoantigen peptide pool was the highest in combined therapy group (Fig. 4K, L), suggesting that the combined therapy induces a strong neoantigen-specific immune response in mice. To further confirm whether potential neoantigen-specific T cells successfully infiltrate the tumor tissue, a fluorescently labelled tetramer for the highly immunogenic neoantigen peptide Mapkbp1 199–206 (RHIKFCYL): H-2 Kb (Mapkbp1) was generated to detect T cells expressing Mapkbp1-specific T cell receptors (TCRs) in the peripheral blood mononuclear cells (PBMCs) and TILs (Fig. 4M–P). Peptide-MHC tetramer staining showed that significantly higher percentages(10.87% ± 0.3786 and 2.797% ± 0.6067) of Mapkbp1 specific CD8^+^ T cells were observed in PBMCs and TILs from mice treated with combined therapy compared to other treated mice (PBS: 0.8033% ± 0.3225 and 0.61% ± 0.2227; LLCvac: 6.850% ± 0.4618 and 1.707% ± 0.157; Bev + anti-PD-1:0.7467% ± 0.1002 and 1.173% ± 0.157, respectively), suggesting the strong neoantigen-specific T-cell infiltration into the tumor by the combined therapy. Overall, these findings suggested that LLCvac plus Bev and anti-PD-1 antibody could elicit strong neoantigen-specific anti-tumor responses for NSCLC treatment.

### Combined treatment enhancing the targeted tumor killing of T cells

**Fig. 5:**
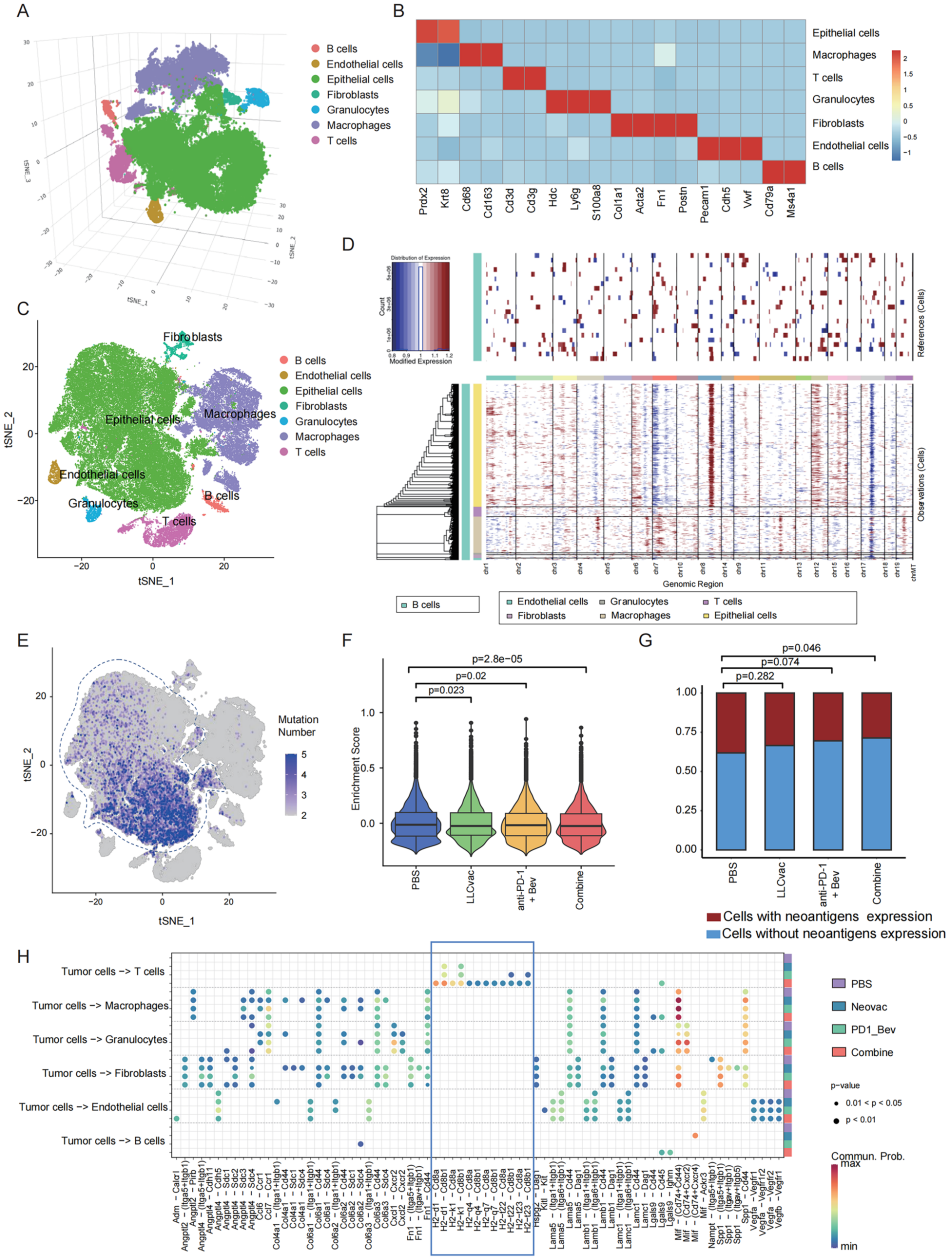
Single-cell RNAseq revealed alterations of LLC tumor tissue characteristics during immunotherapy. **A** The three-dimensional t-distributed stochastic neighbor embedding (t-SNE) plot showing the 11 identified cell clusters within the LLC tumor. **B** Heatmap showing the expression levels of cell type-specific marker genes across each cell cluster. **C** The two-dimensional t-distributed stochastic neighbor embedding (t-SNE) plot showing the 11 identified cell clusters within the LLC tumor. **D** Heatmap of copy number variations (CNVs) for all cell clusters, with B cells as the reference. Amplifications (red) or deletions (blue) were determined by the InferCNV software. **E** t-SNE plot showing the number of somatic mutations detected among all cell clusters. **F** The violin plot showing enrichment score of the parent genes of 7 neoantigen mutations used for neoantigen vaccines preparation at the tumor single cell level across different treatment groups as indicated. **G** Proportion of tumor cells with detected mutant alleles of 3 neoantigen mutation expression (Metap1_T84S, Kat2a_V192I and Dhrs9_ L146P) in different treatment groups. Only cells with read covering at least one of these neoantigen mutation sites were included. **H** Significant ligand-receptor interactions between tumor cell cluster and remaining cell clusters computed using Cellchat

To gain insight into changes in the immune microenvironment of orthotopic LLC models during different therapy, tumors from PBS, LLCvac alone, anti-PD1 + Bev alone, and combined therapy were subjected to single-cell RNA-seq after 20 days of treatment. After quality control, 7 distinct categories of cells, including T cells, epithelial cells, B cells, endothelial cells, fibroblasts, granulocytes and macrophages, were identified (Fig. 5A–C). To determine the tumor cell origin of the epithelial cells, copy number variations were assessed by utilizing B cells as a reference. The inferCNV results revealed that only epithelial cells exhibit frequent amplifications at Chr8, a recurrent genomic event previously reported in lung cancer[18] (Fig. 5D). Moreover, further profiling the distribution of LLC tumor-specific somatic mutations (identified in bulk DNA sequencing data) at single cell level in all cell clusters also revealed that epithelial cell clusters significantly enriched LLC specific somatic mutations, further supporting the tumor essence of this epithelial cell cluster (Fig. 5E).

The neoantigen-specific T cells induced by tumor neoantigen vaccines in vivo could more specifically kill tumor cells that highly express neoantigen mutations. Therefore, we firstly evaluated the expression of parent genes corresponding to those 7 neoantigen mutations used for neoantigen vaccine preparation at the single cell level in identified tumor cells. As shown in Fig. 5F, the signature composed of 7 genes from neoantigen mutations was downregulated in all treatment groups when compared with the PBS group (all *p* < 0.05), with the most significant decrease observed in the combined therapy group (*p* = 2.8 *10^–5^). Subsequently, we further examined the distribution of neoantigen mutation expression among tumor cells in different treatment groups. Due to the limited resolution of single-cell 3' sequencing for mutation detection, only 3 of the 7 neoantigens were detected at the single-cell level, including Metap1_T84S, Kat2a_V192I and Dhrs9_ L146P. Consistently, the combined therapy group exhibited a significant decrease in the proportion of tumor cells expressing those 3 neoantigen mutations when compared to the PBS group (p = 0.046, Fig. 5G), while no statistically significant difference was observed in the other two groups compared to the PBS group. These results suggested that the superior efficacy of the combined therapy may be attributed to the more pronounced elimination of tumor cells expressing the neoantigens.

To further elucidate the major cellular subpopulations involved in neoantigen-based immunotherapy targeting tumor cells, cell–cell communication analysis was performed. As shown in Fig. 5H, only the interactions between T cell subpopulations and tumor cells exhibited different levels of strength among the four treatment groups. The ligand-receptors interactions revealed that the interactions between tumor cells and T cells were mainly focused on the interactions between MHC molecules on tumor cells and CD8 molecules on T cells (such as H2d1-Cd8a, H2d1-Cd8b1, H2k1-Cd8a, H2k1-Cd8b1), which is known as the classical antigen presentation pathway [19]. Moreover, the number of ligand-receptor pairs involved in the antigen presentation pathway between T cells and tumor cells also indicated that the combined therapy exhibited the strongest antigen presentation effect, while the PBS group and the other two treatment groups showed the weakest and intermediate levels of antigen presentation efficacy, respectively (Fig. 5H). Therefore, we inferred that T cell-mediated tumor killing is critical for the therapeutic efficacy of neoantigen-based combined immunotherapy.

### Neoantigen specific CD8_Mki67 T cells increase in tumor immune microenvironment after combined therapy

**Fig. 6:**
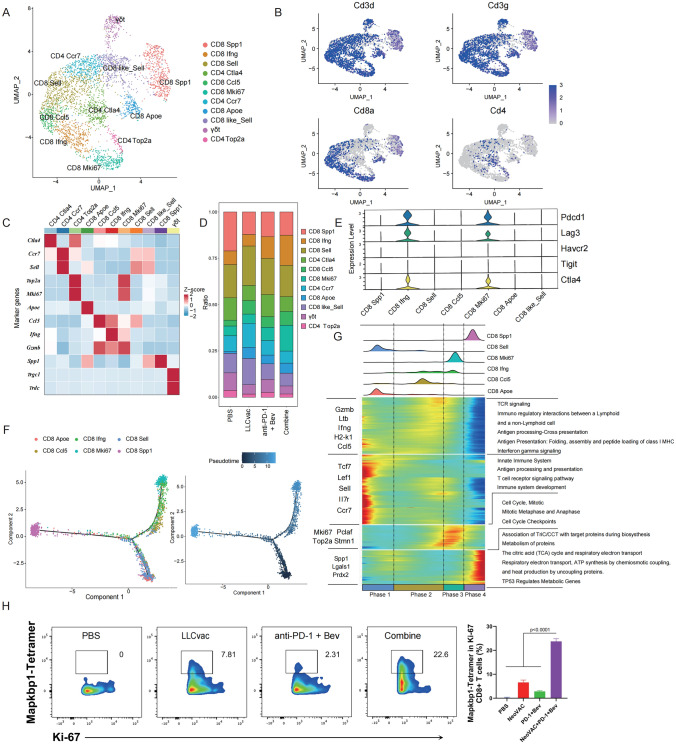
Dynamics of T cells during neoantigen-based immunotherapy. **A** t-SNE plot showing 11 T cell subtypes after T cell re-clustering. **B** t-SNE plot showing expression levels of T cell marker genes. **C** Heatmap illustrating subtype-specific marker gene expression levels across all T cell subtypes. **D** Histogram indicating the proportion of T cell subtypes within each treatment group. **E** Violin plots showing the expression levels of classical immune checkpoints in CD8^+^ T cell subtypes. **F** Pseudotime trajectory of CD8^+^ T cell subtypes, colored by cell subtypes (left); colored by pseudotime (right). **G** The density plot illustrating distribution of different CD8^+^ T cell subtypes along pseudotime (top). Pseudo-heatmap depicting the expression changes of differentially expressed genes across four phases along pseudotime, as well as classical marker genes and pathway enrichment analysis for each phase (bottom). **H** The percentage of neoantigen Mapkbp1-specific T cells in high MKi67 expression of CD8^+^ T cells from tumor tissue in different treatment as indicated

To gain a deeper understanding of the heterogeneity within the T cell compartment in the combined therapy, T cell were extracted and re-clustered to 11 T cell clusters (Fig. 6A). Based on the expression patterns of marker genes (Fig. 6B, C and Fig. S2), 6 clusters of CD8^+^ T cells (CD8_Sell, CD8_Ccl5, CD8_Ifng, CD8_Mki67, CD8_Apoe, CD8_Spp1), three clusters of CD4^+^ T cells (CD4_Ctla4, CD4_Mki67, CD4_Top2a), one cluster of T cells with CD8-like characteristics, and one cluster of Gamma Delta (γδ) T cells were identified. Significantly, upregulation of two cytotoxic CD8^+^ T cell clusters (CD8_Ifng and CD8_Mki67) were observed in the combined therapy group (Fig. 6D). These 2 cell clusters were also co-expressing with activation markers (Pdcd1 and Ctla-4) and effector molecule (Gzmb an Ifng), suggesting the strong proliferative and anti-tumor activity (Fig. 6E). Then we examined the temporal dynamics of these CD8^+^ T cell subsets using the Monocle2 algorithm [20]. Pseudo-heatmap analysis showed that CD8_Sell T cell subset, were at the beginning of the trajectory path, which expresses relatively high levels of naïve T cell markers (Sell, Ccr7) [21], while CD8_Ifng and CD8_Mki67 (upregulated in the combination treatment group) were located at the end of the evolutionary branch on one side, and on the other side are CD8_Spp1 T cells, which were downregulated in the combination treatment group (Fig. 6F). Meanwhile, the change in transcriptional features of those 6 CD8^+^ T cell clusters were further classified into four distinct phases. Among them, CD8_Ifng and CD8_Mki67 are mainly enriched in phage2 and phage 3, which are mainly involved in TCR signaling, immune regulatory interactions between a lymphoid, Cell cycle checkpoints and other signaling pathways (Fig. 6G). These results implied that the combined treatment could induce more naive CD8^+^ T cells to differentiate towards CD8_Ifng and CD8_Mki67 with effector killing ability.

To further validate whether neoantigen-specific T cells are enriched in CD8_ Mki67 cells, we used Mapkbp1 Peptide-MHC tetramer staining to analyze the cell content of neoantigen-specific T in CD8_ Mki67 cells in TILs collected from different treatment groups as indicated. As expected, the content of neoantigen Mapkbp1-specific T cells in high Mki67 expression CD8^+^ T cells was significantly enriched in the combined treatment group than other treatment groups, whereas few neoantigen Mapkbp1-specific T cells were detected in CD8^+^ T cells with low Mki67 expression (Fig. 6H and Fig. S3). Collectively, these results suggest that the combined therapy could significantly increase tumor infiltrating of neoantigen-specific CD8_Mki67 T cells to enhance anti-tumor killing.

## Discussion

Clinically, chemotherapy-free treatment of NSCLC is being welcomed and accepted by more and more patients due to its reduced potential side effects. Therefore, how to improve its anti-tumor therapeutic efficacy without increasing obvious toxicities and side effects is the premise of clinical promotion. The combination of bevacizumab with ICIs has emerged as a first-line promising treatment strategy with high therapeutic efficacy in various solid tumors, including lung and liver cancer [22, 23]. Several studies have shown that the combination of bevacizumab with ICIs significantly increases the infiltration of CD4 and CD8^+^ T lymphocytes within the tumor microenvironment [24, 25]. However, most of these cells are bystander T cells (e.g. influenza virus, EBV virus) with little anti-tumor activity, limiting the improvement in therapeutic efficacy. Neoantigen vaccine can induce the infiltration of tumor-specific T cells without significantly increasing toxicity, and is one of the best choices for clinical combination treatment options. Meanwhile, the tumor mutation burden of NSCLC is high among all tumors, which provided a higher feasibility for patients to screen personalized and high-quality tumor neoantigens for vaccine preparation[26]. Clinical study has confirmed that the combination of first-line treatment with neoantigen vaccine could further improve efficacy and exhibited good safety in NSCLC[15, 16]. In our study, we first demonstrated that the neoantigen vaccine in combination with bevacizumab and ICIs, could significantly improve the anti-tumor efficacy against NSCLC and demonstrate good safety and feasibility. Overall, this approach holds great promise as a new chemotherapy-free strategy for the treatment of NSCLC.

Mki67 is a marker of active cell proliferation. Under the stimulation of tumor antigens, high expression of Mki67 on CD8^+^ T cells can promote their proliferation, further increasing the number of effector cells with anti-tumor effects and has been suggested to be associated with a better prognosis in various solid tumors[27, 28]. TCR tracking in liver cancer, lung cancer, and colorectal cancer has shown that Mki67-positive CD8^+^ T cells share the same TCR sequence (paired α and β chains) with those identified exhausted effector T cells with anti-tumor effects, suggesting that they have the same origin but different states[29]. Additionally, Mki67-positive CD8^+^ T cells may contribute to the formation of immune memory, enabling the cells to better respond to the same antigen upon re-encounter, thereby maintaining long-term anti-tumor immune effects and preventing tumor recurrence and metastasis[30, 31]. Consistently, our research has also shown that combined treatment, including neoantigen vaccines with bevacizumab and anti-PD-1 antibody, can induce the differentiation of naïve CD8^+^ T cells into Mki67-positive T cells within the tumor, leading to increased enrichment of neoantigen-specific Mki67-positive CD8^+^ T cells at the tumor site, indicating potent anti-tumor immune effects.

## Conclusions

In summary, we have established a safe and feasible comprehensive treatment approach for advanced NSCLC. The combination of neoantigen vaccines with bevacizumab and immune checkpoint inhibitors (ICIs) significantly enhances the enrichment of neoantigen-specific Mki67 T cells within tumor, thereby improving anti-tumor immune responses. This combination therapy offered a new chemotherapy-free treatment option for patients who were intolerant or intolerable to chemotherapy. However, due to the limited NSCLC models used in this study, the corresponding findings need to be further validated in other NSCLC animal models and clinical trials.

### Supplementary Information

Below is the link to the electronic supplementary material.

Supplementary file1 (DOCX 5032 KB)

## Data Availability

All data are available in a public, open access repository. The raw sequencing data in this article has been deposited at Genome Sequencing Achieve database (https://ngdc.cncb.ac.cn/) under the accession number of CRA012095.

## Abbreviation

BEV: Bevacizumab
CNV: Copy number variation
DCs: Dendritic cells
ELISPOT: Interferon (IFN)-γ enzyme-linked immunospot
ICIs: Immune checkpoint inhibitors
LLC: Lewis lung carcinoma
LLCvac: Neoantigen peptide vaccine for LLC tumor
MHC: Major histocompatibility complex
NSCLC: Non-small cell lung cancer
PBMCs: Peripheral blood mononuclear cells
PBS: Phosphate buffered saline
PCA: Principal component analysis
UMIs: Unique molecular identifiers
VEP: Variant effect predictor
