# Supplementary material for: Personalized neoantigen vaccine enhances the therapeutic efficacy of bevacizumab and anti-PD-1 antibody in advanced non-small cell lung cancer
Source: Cancer Immunol Immunother. 2024 Jan 27;73(2):26. doi: 10.1007/s00262-023-03598-x (PMC10821847; doi:10.1007/s00262-023-03598-x)
Supplement: Supplementary file 1 — Supplementary file1 (DOCX 5032 KB) [file 262_2023_3598_MOESM1_ESM.docx]

**Personalized** **neoantigen vaccine enhances** **the** **therapeutic efficacy of** **bevacizumab and anti PD-1 antibody in advanced** **non-small cell lung cancer**

Xiuhua Lin^1,2*^, Shichuan Tang^2,3*^, Yutong Guo^2,3*^, Ruijing Tang^2,3*^, Zhenli Li^2,3^, Xinting Pan^2,3^, Geng Chen^2,3^, Liman Qiu^2,3^, Xiuqing Dong^2,3^,Ling Zhang^5,6^, Xiaolong Liu^2,3^, Zhixiong Cai^2,3,^**^#^**, Baosong Xie^1,4,^**^#^**

1. Shengli Clinical Medical College of Fujian Medical University, Fuzhou, China

2.The United Innovation of Mengchao Hepatobiliary Technology Key Laboratory of Fujian Province, Mengchao Hepatobiliary Hospital of Fujian Medical University, Fuzhou, China

3.The Liver Center of Fujian Province, Fujian Medical University, Fuzhou, China

4. Department of Pulmonary and Critical Care Medicine, Fujian Provincial Hospital, Fuzhou, China

5. Academy of Integrative Medicine, Fujian University of Traditional Chinese Medicine, 1 Qiuyang Road, Minhou Shangjie, Fuzhou, China;

6. Fujian Key Laboratory of Integrative Medicine on Geriatrics, Fujian University of Traditional Chinese Medicine, 1 Qiuyang Road, Minhou Shangjie, Fuzhou, China;

^*^Both authors contributed equally to this work.

**^#^ Corresponding authors:**

Cai, Z., caizhixiong1985@163.com, ORCID: 0000-0002-0912-8372

The United Innovation of Mengchao Hepatobiliary Technology Key Laboratory of Fujian Province, Mengchao Hepatobiliary Hospital of Fujian Medical University, Xihong Road 312, Fuzhou 350025, Fujian Province, China.

Xie, B. , xbaosong@126.com, ORCID:0000-0002-4948-0063

Shengli Clinical Medical College of Fujian Medical University, East Street 134, Fuzhou 350001, Fujian Province, China.

**Supplementary Methods**

**Neoantigen identification and immunogenicity validation**

To identify potential neoantigens derived from Lewis lung carcinoma (LLC) tumors, we performed whole exome sequencing and transcriptomic sequencing on the LLC cell line and C57BL/6 mouse tail tissue. The detailed methods have been described in previously published literature([1](#_ENREF_1)). The exome sequencing data were aligned to the mouse genome (mm10) using the BWA algorithm ([2](#_ENREF_2)). Duplicate reads were then identified and removed using Picard ([3](#_ENREF_3)). Subsequently, somatic mutations were called using Mutect2([4](#_ENREF_4)) with C57BL/6 mouse tail tissue serving as the normal reference. All identified somatic mutations were further validated by bam2R algorithm from R package DeepSNV and the underwent annotation with Variant Effect Predictor (VEP) ([5](#_ENREF_5)), and missense mutations were tested for immunogenicity examination using the pVAC-Seq pipeline ([6](#_ENREF_6)). For each missense mutation, the binding affinity of 8~11 mer peptides containing mutated amino acids to H2K^b^ was predicted using a comprehensive set of algorithms, including NetMHCpan, NetMHC, NetMHCcons, PickPocket, MHCflurry, SMM, SMMPBMC and MHCnuggetsI. The transcriptome sequencing data were aligned to the mouse genome (mm10) using the STAR aligner ([7](#_ENREF_7)), together with the GENCODE gene annotation. The expression levels of all genes were then quantified using the RSEM algorithm ([8](#_ENREF_8)), which calculates the metric of transcripts per million (TPM) metric. Finally, by combining information from both DNA and RNA sequencing data, mutations fulfilling the following criteria were considered as candidate neoantigens: (1) mutations with median binding affinity percentile rank across all eight MHC binding affinity prediction algorithms (NetMHCpan, NetMHC, NetMHCcons, PickPocket, MHCflurry, SMM, SMMPBMC and MHCnuggetsI) <=2%; (2) mutations with >= 20× depth and variant allele frequency (VAF) >= 0.1 in LLC tumor cells at both DNA and RNA level; (2) mutations with >= 20× depth and variant allele frequency (VAF) <= 0.01 in normal reference; (4) mutations located at genes and transcripts with TPM >=1; (5) mutations with DNA frequency <=0.6 (in order to exclude possible germline mutations).

Then top 20 potential neoantigen mutations were selected and prioritized for long peptide synthesis (17 amino acids in length) using standard solid-phase synthetic peptide chemistry ( >95% purity, Jinsirui Biotechnology, China). For neoantigen immunogenicity validation, 16 successfully synthesized candidate neoantigen peptides were randomly divided into two pools (100µg/peptide) and mixed with 50µl Poly(I:C) (Guangdong South China Pharmaceutical), then injected subcutaneously in the lateral flank of 2 groups of C57BL/6 mice on days 0, 4 and 8, respectively. Mice were sacrificed on day 14 and splenic T cells were harvested for ELISPOT assay according to the manufacturer's instructions.

**Enzyme-linked immunospot (ELISPOT) assay**

IFN-γ secretion of mouse splenic T cells were detected by ELISPOT kit (Mabtech，3321-4APT-10). The detailed methods have been described in previously published literature([1](#_ENREF_1)). Briefly, bone marrow derived-DCs (BMDCs) were isolated from femurs and tibias from 6-8 weeks old naive C57BL/6 mice. After removal of residual soft tissue and epiphyses, the marrow was collected by flushing the canals with PBS and centrifuged at 800g for 5min at room temperature. The precipitated cells were lysed with 1mL Red Blood Cell Lysis Solution (Gibco) for 4 minutes, centrifuged at 800g for 5 minutes, and washed twice with PBS. At day 0, 2 million cells/well were added into a 6-well plate and cultured with 2 mL RPMI-1640 medium/well (10 ng/mL IL-4, 404-ML-010/CF and 20 ng/mL mGM-CSF, R&D systems, 415-ML-020/CF) to obtain BMDCs at 37°C with 5% CO_2_. Half volume of medium was changed at day 3. At day 6, BMDCs were pulsed with neoantigen peptide pool (4µg in total，0.55µg per peptide) or each peptide (4µg) for 48h. For ELISPOT assay, 3×10^4^ BMDCs (pulsed with neoantigen peptide as described previously) were co-incubated with 3×10^5^ splenic T cells in a multiscreen 96-well filtration plate (Mabtech，3321-4APT-2) at 37℃ with 5% CO_2_ for another 48h. BMDCs pulsed with PBS were used as a negative control, while splenic T cells co-incubated with CD3 antibody for T cell activation were used as a positive control. The plates were washed and subsequently incubated with detection antibody (R4-6A2-biotin,1 µg/mL, 100µl/well) for 2 hours at room temperature. The plates were washed again and then incubated with Streptavidin ALP (1:1000 dilution, 100µl per well) for 1 hour at room temperature. Subsequently, 3, 3', 5, 5'-T etramethylbenzidine (TMB) substrate solution was added to each well and incubated for 4-8 min at room temperature before adding deionized water to stop the reaction. Finally, IFN-γ spot-forming cells were imaged and analyzed by ELISPOT Analysis System (AT-Spot-2200, Beijing Antai Yongxin Medical Technology Co., Ltd). A score of IFN-γ spot-forming cells above 50 is considered positive.

**Flow cytometry**

Single cell suspensions from lymph nodes, spleen and tumors were stained with different combinations of antibodies, including anti-mouse CD11c-APC mAb (eBioscience), anti-mouse CD80-PE mAb (eBioscience) and anti-mouse CD86-PE-Cyanine7 mAb (eBioscience), anti-mouse CD8-PE/FITC mAb (eBioscience), anti-mouse CD44-PE-Cyanine7 mAb (eBioscience), anti-mouse CD62L-PerCP/Cy5.5 mAb (eBioscience), anti-mouse CD3-APC/FITC mAb (eBioscience), and anti-mouse CD4-PE mAb (eBioscience).

The anti-mouse peptide-specific tetramer-PE was generated using the QuickSwithTM Quant Tetramer Kit (MBL international, TB-7400-K1) as previously described([1](#_ENREF_1)). Briefly, it was generated by mixing 50 μl of tetramer (50 μg/mL) with 1μl of peptide solution (10 nM) and 1μl of proprietary peptide exchange factor and incubated for 4h at room temperature in the dark. Tumor single cell suspensions were stained with 2μl tetramer for 30 minutes at room temperature, then anti-mouse CD8 antibody was added and incubated for another 20 minutes at room temperature. All samples were run on a flow cytometer (BD FACSVerseTM, USA) and data were analysed by FlowJo v.10.

**HE staining**

HE staining was performed according to routine protocols. Briefly, after deparaffinisation and rehydration, organ sections were stained with hematoxylin solution (Servicebio, G1004) for 5 minutes, followed by 5 dips in 1% acid ethanol (1% HCl in 75% ethanol) and then rinsed in distilled water. Then the sections were stained with eosin solution (Servicebio, G1001) for 3 minutes and followed by dehydration with graded alcohol and clearing in xylene. The HE-stained slides were digitised, and crop images were collected.

**Immunofluorescence**

Tumor tissue sections were identical to the HE-stained sections. Primary antibodies [rabbit anti-mouse CD4 mAb (Servicebio, GB13064-1), and rabbit anti-mouse CD8 mAb (Servicebio, GB13068)] were added after successful blocking and incubated for 12h at 4°C in a refrigerator, followed by secondary antibody incubation for 30 minutes at room temperature. After staining (2 randomly selected from each group), the sections were digitized at 10/40 magnification (objective lens) and crop images were captured by fluorescence microscopy.

**Single-cell RNA sequencing of LLC tumor tissue**

Fresh tumor tissues were collected from LLC mouse models treated with LLCvac and/or BeV plus anti-PD1 antibody for 20 days, as well as a PBS-treated control group. The tissues were immediately placed on ice and digested with 2 mL of sCelLiveTM Tissue Dissociation Solution (Singleron, China). The resulting cells were then suspended in PBS. Cell viability was assessed microscopically using the trypan blue exclusion test (Gibco, Grand Island, NY, USA), and samples with cell viability greater than 80% were considered suitable for subsequent experiments. The cell suspension was further diluted in PBS to achieve a concentration of 3×10^5 cells/ml. Single cell isolation and mRNA capture were performed using the Singleron Matrix Single Cell Processing System (Singleron, China). Subsequently, scRNA-seq libraries were constructed by reverse transcription, amplification, and library construction using the GEXSCOPE Single Cell RNA Library Kit (Singleron, China). Only libraries that met quality standards were sequenced on the Illumina HiSeq 6000 platform (150bp paired-end reads).

**Single-cell RNA sequencing data analysis.**

Raw single-cell RNA sequencing data were processed using the CeleScope pipeline (<https://github.com/singleron-RD/CeleScope/,v1.12.0>) with default parameters. Briefly, low quality reads were removed and the remaining qualified reads were mapped to the mouse reference genome GRCm38 (Ensembl release 99) after adapter and poly(A) tail trimming. Following the extraction of unique molecular identifiers (UMIs) and barcodes, the number of UMIs mapped to each annotated gene for each cell barcode was counted to generate an expression matrix consisting of UMI counts, providing a consistent representation of gene expression for subsequent analyses.

Based on the UMI matrix obtained from the four single-cell libraries, the data were further merged and filtered with Seurat (v4.3.0)^(^[^9^](#_ENREF_9)^)^ in R (v4.1.3) using following criteria: genes detected in fewer than 3 cells; cells with fewer than 200 genes detected; cells with more than 5000 genes detected; cells with more than 15% of UMI reads mapped to mitochondrial genes; cells with detected numbers of genes or UMIs exceeding the 98th percentile of all cells in the dataset. After excluding the low-quality cells, the combination therapy group retained 17293 cells, the neoantigen vaccine treatment alone group retained 20322 cells, the α-PD1 treatment alone group retained 18905 cells, and the control group retained 15335 cells.

Within the Seurat framework, the filtered gene expression data was normalized using the NormalizeData function, employing the LogNormalize method. Subsequently, the FindVariable function was used to identify the top 2000 variable genes. The gene expression data was then scaled using the ScaleData function and principal component analysis (PCA) was performed. The Harmony algorithm (v0.1.1) was used to better integrate the four samples. A t-SNE dimensionality reduction was performed, followed by cell clustering using the Seurat functions FindNeighbors and FindClusters. Then cell clusters were annotated by SingleR ([10](#_ENREF_10)) package (v1.8.1) using the annotation from MouseRNAseq data set ([11](#_ENREF_11)).

The cluster annotations were further confirmed by the expression of the following canonical markers: Endothelial cells (Pecam1, Cldn5, Vwf), Fibroblasts (Col1a1, Acta2, Fen1, Postn), T cells (CD3D, CD3G), B cells (Cd79a, Ms4a1), granulocytes (Hdc, Ly6g, S100a8), macrophages (Cd68, Cd163), and epithelial cells (Krt8, Prdx2). The tumor nature of the epithelial cell cluster was further confirmed by the presence of frequent genomic alterations, including copy number variations at the single cell level and somatic mutations.

**Single-cell RNAseq-based CNVs detection**

Copy number variation (CNV) patterns within each cell cluster were inferred using InferCNV ([12](#_ENREF_12)) (v1.14.2) based on single cell transcriptomic profiles. Using B cells as a reference, we applied the denoise mode, utilized hidden Markov model (HMM) settings, and set the "cutoff" parameter to 0.1. To ensure both rigour and computational resources, we randomly selected 1000 qualified cells and performed the InferCNV analysis three times.

**Single-cell RNAseq-based mutation identification**

Somatic mutation detection at the single cell level was performed using cellsnp-lite software (v1.2.2) ([13](#_ENREF_13)). Briefly, we used single-cell RNA-seq BAM files, which were obtained after mapping, along with VCF files containing somatic mutations derived from bulk DNA sequencing data, as input for the analysis. The somatic mutations from bulk DNA were called using Mutect2, as described above, and mutations of high quality (>= 20× depth in both LLC tumor and normal reference, VAF>=0.1 and VAF<=0.6 in LLC tumor, and VAF < 0.01 in the normal reference) were used for mutation detection at the single cell level. The cellsnp-lite analysis generates two sparse matrixes containing the information of variant allele depth and total sequencing depth for each cell at each variant position. Then the number of somatic mutations or ELISPOT-positive neoantigens detected at the single cell level was calculated for further comparisons.

**Cell-cell interaction analysis**

To identify potential cell clusters that interact with tumor cell cluster and affected by immunotherapy, we employed Cellchat([14](#_ENREF_14)) to identify significant cell-cell interactions. We designated the tumor cell cluster as the "target" and considered ligand-receptor pairs with a p-value < 0.05 as significant interactions. This analysis was performed separately for each group and the results were combined for comparative analysis.

**CD8^+^ T cell developmental trajectory**

The cell differentiation trajectory of CD8+ T cells was reconstructed using the Monocle2 algorithm (v2.22.0) ([15](#_ENREF_15)). After extracting the expression matrix of CD8+ T cell clusters from the Seurat object, genes expressed in less than 10 cells were filtered out. The top 2000 highly variable genes identified by the 'differentialGeneTest' function with a q-value < 0.001 were utilized to order cells in the pseudotime analysis.

**Pathway enrichment analysis**

The differentially expressed genes along the CD8^+^ T cell pseudo time trajectory of were extracted and subjected to Reactome enrichment analysis using the ConsensusPathDB tool ([16](#_ENREF_16)). To evaluate the enrichment of ELISPOT-positive genes at the single cell level, a gene signature was constructed, and the "AddModuleScore" function in Seurat was utilized to calculate the enrichment score for each individual cell.

**References**

1. Chen H, Li Z, Qiu L et al. (2022) Personalized neoantigen vaccine combined with PD-1 blockade increases CD8+ tissue-resident memory T-cell infiltration in preclinical hepatocellular carcinoma models. Journal for ImmunoTherapy of Cancer. 10: e004389. doi: 10.1136/jitc-2021-004389

2. Li H, Durbin R (2009) Fast and accurate short read alignment with Burrows-Wheeler transform. Bioinformatics. 25: 1754-60. doi: 10.1093/bioinformatics/btp324

3. DePristo MA, Banks E, Poplin R et al. (2011) A framework for variation discovery and genotyping using next-generation DNA sequencing data. Nat Genet. 43: 491-8. doi: 10.1038/ng.806

4. Cibulskis K, Lawrence MS, Carter SL et al. (2013) Sensitive detection of somatic point mutations in impure and heterogeneous cancer samples. Nat Biotechnol. 31: 213-9. doi: 10.1038/nbt.2514

5. McLaren W, Gil L, Hunt SE, Riat HS, Ritchie GR, Thormann A, Flicek P, Cunningham F (2016) The Ensembl Variant Effect Predictor. Genome Biol. 17: 122. doi: 10.1186/s13059-016-0974-4

6. Hundal J, Carreno BM, Petti AA, Linette GP, Griffith OL, Mardis ER, Griffith M (2016) pVAC-Seq: A genome-guided in silico approach to identifying tumor neoantigens. Genome Med. 8: 11. doi: 10.1186/s13073-016-0264-5

7. Dobin A, Davis CA, Schlesinger F, Drenkow J, Zaleski C, Jha S, Batut P, Chaisson M, Gingeras TR (2013) STAR: ultrafast universal RNA-seq aligner. Bioinformatics. 29: 15-21. doi: 10.1093/bioinformatics/bts635

8. Li B, Dewey CN (2011) RSEM: accurate transcript quantification from RNA-Seq data with or without a reference genome. BMC Bioinformatics. 12: 323. doi: 10.1186/1471-2105-12-323

9. Satija R, Farrell JA, Gennert D, Schier AF, Regev A (2015) Spatial reconstruction of single-cell gene expression data. Nat Biotechnol. 33: 495-502. doi: 10.1038/nbt.3192

10. Aran D, Looney AP, Liu L et al. (2019) Reference-based analysis of lung single-cell sequencing reveals a transitional profibrotic macrophage. Nat Immunol. 20: 163-72. doi: 10.1038/s41590-018-0276-y

11. Benayoun BA, Pollina EA, Singh PP, Mahmoudi S, Harel I, Casey KM, Dulken BW, Kundaje A, Brunet A (2019) Remodeling of epigenome and transcriptome landscapes with aging in mice reveals widespread induction of inflammatory responses. Genome Res. 29: 697-709. doi: 10.1101/gr.240093.118

12. Patel AP, Tirosh I, Trombetta JJ et al. (2014) Single-cell RNA-seq highlights intratumoral heterogeneity in primary glioblastoma. Science. 344: 1396-401. doi: 10.1126/science.1254257

13. Huang X, Huang Y (2021) Cellsnp-lite: an efficient tool for genotyping single cells. Bioinformatics. 37: 4569-71. doi: 10.1093/bioinformatics/btab358

14. Jin S, Guerrero-Juarez CF, Zhang L, Chang I, Ramos R, Kuan CH, Myung P, Plikus MV, Nie Q (2021) Inference and analysis of cell-cell communication using CellChat. Nat Commun. 12: 1088. doi: 10.1038/s41467-021-21246-9

15. Qiu X, Mao Q, Tang Y, Wang L, Chawla R, Pliner HA, Trapnell C (2017) Reversed graph embedding resolves complex single-cell trajectories. Nat Methods. 14: 979-82. doi: 10.1038/nmeth.4402

16. Kamburov A, Stelzl U, Lehrach H, Herwig R (2013) The ConsensusPathDB interaction database: 2013 update. Nucleic Acids Res. 41: D793-800. doi: 10.1093/nar/gks1055

**Supplementary Information**

**Figure S1:** The changes of biochemical indicators in mice receiving different treatments as indicated.

**Figure S2:** Violin plots showing the expression level of Cd8a, Cd8b1 and Cd4 for each T cell subtype.

**Figure S3:** The percentage of neoantigen Mapkbp1-specific T cells in different MKi67 expression of CD8 T cells from tumor tissue in combined therapy.

**Table S1.** Synthesized candidate LLC neoantigen peptides with high affinity to H-2Kb alleles

**Figure S1**


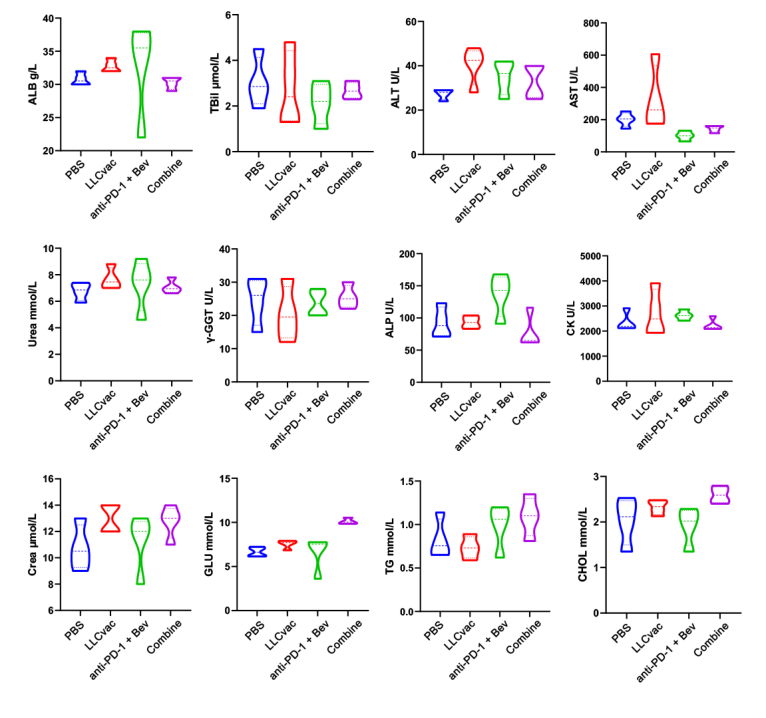


**Figure S2**


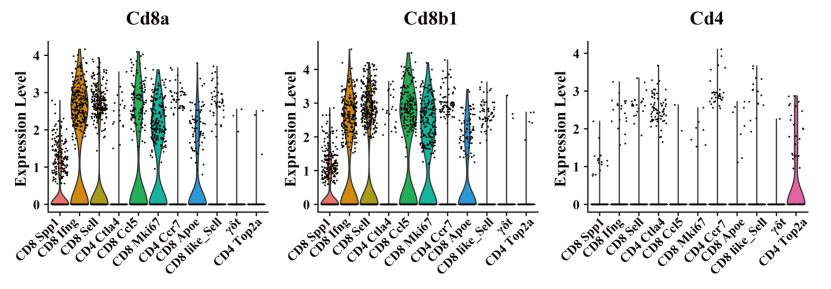


**Figure S3**


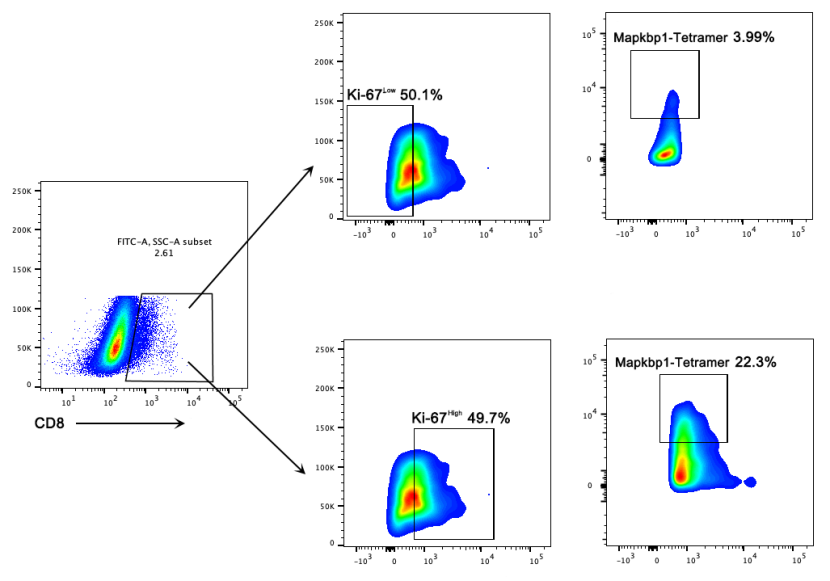


**Table S1. Synthesized candidate LLC neoantigen peptides with high affinity to H-2K^b^ alleles**

| **ID** | **Gene** | **Mutation** | **Expression level (TPM^a^)** | **Peptide (MT^b^)** | **Median.MT. Percentile(%)** |
| --- | --- | --- | --- | --- | --- |
| chr11_35783472 | Pank3 | Gly321Trp | 22.85 | KINRVVFVWNFLRVNTL | 0.09 |
| chr9_107583356 | Naa80 | Gly150Ala | 4.7 | PGAAPVVVAHARLSRVL | 0.1 |
| chr1_62771792 | Nrp2 | Trp664Leu | 4.06 | GWVYDHAKLLRSTWISS | 0.223 |
| chr5_138101361 | Zkscan1 | Lys522Arg | 6.62 | PYKCTKCGRAFTRSSTL | 0.24 |
| chr2_157806691 | Ctnnbl1 | Ala194Val | 3.53 | DALVDGQVVALLVQNLE | 0.27 |
| chr5_138133441 | Zscan21 | His409Leu | 1.28 | NLTLHYRTLLVDRPYDC | 0.27 |
| chr1_95325147 | Fam174a | Val158Ala | 3.06 | NRKTRRYGALDTNIENM | 0.29 |
| chr3_138480716 | Metap1 | Thr84Ser | 18.01 | DPWAGYRYSGKLRPHYP | 0.36 |
| chr5_122458097 | Atp2a2 | Ala911Gly | 38.39 | LVTIEMCNGLNSLSENQ | 0.39 |
| chr11_100711353 | Kat2a | Val192Ile | 10.59 | EEDTDTKQIYFYLFKLL | 0.4 |
| chr2_69394406 | Dhrs9 | Leu146Pro | 4.34 | LFGLINVTPNMLPLVKK | 0.44 |
| chr18_7910758 | Wac | Ala240Thr | 15 | KKSFDANGTSTLSKLPT | 0.462 |
| chr2_120012703 | Mapkbp1 | Trp204Cys | 3.37 | AGNRHIKFCYLDDSKTS | 0.478 |
| chr2_80540580 | Nckap1 | Lys344Thr | 28.48 | GSMHRERRTFLRSALKE | 0.509 |
| chr5_108092153 | Mtf2 | Asp242Ala | 4.84 | LQKPMLFGARFYTFICS | 0.66 |
| chr15_100183054 | Dip2b | Val859Ala | 22.85 | VFYDERIVAVAEQRPDA | 0.68 |

a: TPM, transcripts per kilobase per million mapped reads;

b: MT, mutation
